# Normoxic Oxygen at Birth Enhances Piglet Growth and Survival Under Tropical Farm Conditions

**DOI:** 10.3390/ani16010111

**Published:** 2025-12-30

**Authors:** Phoo Pwint Pwint Thu, Rafa Boonprakob, Padet Tummaruk, Roy Kirkwood, Nutthee Am-In

**Affiliations:** 1 Department of Obstetrics, Gynaecology, and Reproduction, Faculty of Veterinary Science, Chulalongkorn University, Bangkok 10330, Thailand; phoo.ppthu28@gmail.com (P.P.P.T.); padet.t@chula.ac.th (P.T.); 2 Department of Quality Assurance and Animal Health Office, Livestock Production Betagro Group, Bangkok 10210, Thailand; rafab@betagro.com; 3 Centre of Excellence in Swine Reproduction, Chulalongkorn University, Bangkok 10330, Thailand; 4 School of Animal and Veterinary Sciences, Adelaide University, Roseworthy Campus, Roseworthy, SA 5371, Australia; roy.kirkwood@adelaide.edu.au

**Keywords:** colostrum intake, oxygen supplementation, neonatal piglets, vitality, glucose, survival, tropical conditions

## Abstract

Neonatal piglet mortality remains a major challenge in hyper-prolific sow herds, particularly under tropical conditions where low ambient oxygen (18–21%) may exacerbate hypoxia during prolonged farrowing. This study evaluated whether restoring normoxia (21% O_2_) immediately after birth improved piglet colostrum intake, blood glucose levels, and survival to 3 d. A total of 1837 piglets from 95 sows were weighed at birth and assigned by litter to exposure to either a normoxic chamber (FiO_2_ = 0.21) or ambient-air control (FiO_2_ = 0.16–0.18) for 15 min post-partum. Piglet colostrum intake, blood glucose content, and survival to 24-h and 3 d were analyzed using mixed-effects models. Compared with controls, oxygen-supplemented piglets showed higher postnatal oxygen saturation (94.2 ± 3.6 vs. 88.1 ± 3.5%), greater colostrum intake (348 ± 263 vs. 320 ± 233 g), higher blood glucose (97.7 ± 13.0 vs. 93.8 ± 13.1 mg/dL), and increased 3-day survival (86.1% vs. 80.8%; *p* < 0.01). The effect was most pronounced in piglets < 1.0 kg and >1.3 kg, reflecting improved recovery from transient hypoxia. Mean weaning weight increased by 0.3 kg, indicating sustained benefits in early growth. These findings demonstrate that short-term normoxic oxygen supplementation is a practical, cost-effective intervention that enhances growth, metabolic stability, and survival in neonatal piglets under tropical commercial farm conditions.

## Simple Summary

Modern sows often give birth to large litters, which can prolong farrowing and cause oxygen shortages in newborn piglets. Piglets that do not get enough oxygen at birth are weak, suckle poorly, and have a higher risk of death. This field study tested a simple remedy, giving newborn piglets normal air containing 21% oxygen for 15 min right after birth. The study included almost 1800 piglets from a commercial farm in Thailand. Piglets that received oxygen showed higher blood oxygen levels, drank more colostrum, had higher blood sugar, and were more likely to survive the first three days. The results show that restoring normal oxygen levels is a practical and low-cost way to improve piglet vitality and survival in tropical farms.

## 1. Introduction

Despite advances in hyper-prolific sow genetics, neonatal piglet mortality remains a major challenge in modern swine production, with 15–20% of piglets dying during lactation or farrowing [1,2]. Tropical farm conditions, including those in Thai commercial swine systems, are characterized by persistently high temperatures and relative humidity typically ranging from 70–85%. Although evaporative cooling systems are widely used, they reduce but do not normalize barn temperatures, particularly during farrowing. In contrast to the more stable environments of temperate regions, these tropical conditions impose sustained heat load that can impair farrowing performance and reduce piglet survival during the early postnatal period. The first 72 h postpartum represent a critical window, primarily due to low birth weights, hypothermia, starvation, crushing, and insufficient colostrum intake [3,4]. Previous studies report that approximately 20% of piglets are stillborn or die shortly after birth. Stillbirths are commonly associated with intrapartum hypoxia, particularly during prolonged farrowing, which impairs oxygen delivery to the fetus [5]. Pre-weaning mortality (PWM) remains a major concern, with reported rates of around 20% in the Netherlands and Thailand, 18–23% in Denmark and 11–21% in Sweden and Switzerland, largely associated with low birth weight, hypothermia, inadequate colostrum intake, and management practices [6,7,8]. In contemporary pig production, hyper-prolific sows frequently give birth to litters larger than 16 piglets; this characteristic is associated with prolonged farrowing (>240–300 min) [9], which increases the number of uterine contractions, reduces fetal oxygen supply, and compromises placental blood flow [10,11].

For newborn metabolism, thermoregulation, and organ function, adequate oxygenation is essential because oxygen promotes aerobic adenosine triphosphate (ATP) synthesis and energy balance, whereas hypoxia hinders glucose metabolism and restricts energy mobilization, which weakens a piglet’s capacity to nurse and regulate their body temperature [12]. Piglets experiencing neonatal hypoxia typically show impaired neuromuscular function and poor suckling ability, which limits colostrum intake, early growth and, ultimately, viability [13,14]. Inadequate colostrum not only restricts energy and passive immunity but also heightens risks of infection, hypoglycemia, and thermoregulatory failure, thereby increasing pre-weaning mortality [15,16].

Oxygen therapy has been investigated as a supportive intervention in neonatal piglets, yet the outcomes remain inconsistent. A 24 h period of constant 100% oxygen exposure resulted in lung damage that included inflammation, epithelial damage, and compromised pulmonary function [17]. In addition, resuscitation with high oxygen concentrations (100%, 60%, or 40%) was found to be detrimental compared to room air after global hypoxia [18]. In contrast, short-term oxygen supplementation at birth improved vitality, colostrum intake, and survival [19,20], but long-term supplementation after hypoxia can affect brain oxygenation and dopamine metabolism [21,22]. These conflicting results highlight the importance of conducting effectively organized field research to ascertain an appropriate oxygen therapy concentration and duration to maximize advantages and minimize oxidative damage.

In our commercial setting, ambient farrowing-house oxygen was consistently 16–18%, which is below normal atmospheric levels (~20.9%). This can exacerbate perinatal hypoxia in neonates, particularly following prolonged farrowing and delivery of low-birth-weight litters. We therefore framed the intervention as normoxic air supplementation, restoring the fractional inspired oxygen to FiO_2_ = 0.21, rather than high-dose oxygen therapy. This choice aimed to relieve hypoxia, while avoiding hyperoxia, which has been linked to oxidative stress and pulmonary/neurologic injury when delivered for prolonged periods [17,18,21,22]. This is consistent with human neonatology, where room-air resuscitation achieves outcomes comparable to 100% oxygen with less oxidative burden [23]. Previous research shows that short, targeted oxygen supplementation immediately after birth can improve piglet vitality and early survival [20], while colostrum intake is another major factor influencing early neonatal outcomes [24]. Selecting 21% O_2_ is thus physiologically sound, isolates the effect of oxygen independent of heat/handling, and is readily implementable on farm. We hypothesized that restoring FiO_2_ from 16–18% to 0.21 for 15 min immediately after birth would increase SpO_2_, enhance colostrum intake and glycemic stability, and improve 3-day survival, with the greatest benefit expected among low-birthweight.

Although oxygen treatment is being used more in the treatment of neonatal piglets, little is known about how it affects nutrient utilization, colostrum consumption, and survival. Limited research has directly assessed pre-oxygen saturation levels (SpO_2_B) and post-oxygen saturation levels (SpO_2_A) supplementation, and even fewer studies have related these measurements to colostrum consumption, metabolic processes, and commercial survival. Although the significance of colostrum consumption and oxygenation has each been acknowledged, there is a dearth of data concerning their interaction. Most previous research has relied on indirect indicators of vitality rather than objective physiological measurements, limiting the ability to establish oxygen requirements or design targeted interventions. Accordingly, the present work aimed to investigate how immediate postnatal administration of 21% oxygen influenced piglet oxygenation status, colostrum intake, blood glucose concentration, and survival at 24 h and 3 d of age.

## 2. Materials and Methods

### 2.1. Animals and Housing

This study was conducted on a commercial swine herd located in northern Thailand. A total of 95 healthy Landrace × Yorkshire crossbred sows (parity 2–7) and their 1837 piglets were enrolled. All sows originated from the same breeding unit and were managed under standard commercial practices. At 109 d of gestation, sows were moved to individual farrowing pens equipped with creep heating lamps. Farrowing-room temperature was maintained at 25–29 °C using evaporative cooling ventilation and fans, representing typical tropical commercial conditions. However, this range exceeds the recommended thermal comfort zone for farrowing sows (18–25 °C), which may contribute to environmental heat load [25,26]. Each farrowing pen had a heating lamp over the creep area for piglets, but to help dry the piglets and minimize a drop in body temperature following birth, an additional heat lamp was temporarily placed above the sow during delivery. This process was closely monitored and stopped once the piglets were dry to prevent too much heat exposure. Standard farm management practices, including sow feeding (per NRC guidelines), drying of piglets, and umbilical cord ligation, were applied as per farm protocol. Stillborn piglets and those with visible congenital malformations or severe injuries at birth were excluded from physiological and survival analyses. All animal procedures were approved by the Chulalongkorn University Institutional Animal Care and Use Committee (Animal Use Protocol No. 2431025) in accordance with relevant guidelines and regulations. Throughout the study, data were collected by trained personnel under the supervision of a farm veterinarian.

### 2.2. Experimental Design

Entire litters were allocated at the sow level (*n* = 95) to one of two treatments: a normoxic chamber in which the fractional inspired oxygen (FiO_2_) was stabilized at 0.21, or an ambient control chamber supplied with untreated farrowing-house air maintained at 0.16–0.18 FiO_2_. Allocation to treatment was stratified by sow parity and expected farrowing date to balance groups, and all piglets within each litter received the same assigned exposure immediately after birth. The allocation was performed strictly at the sow (litter) level to prevent cross-contamination or handling bias between treatments, and no piglet from one litter was mixed or exchanged with another during the study. Outcome assessors were blinded to treatment allocation.

### 2.3. Oxygen Chamber Treatment

Immediately after birth, piglets were allocated to treatment according to their dam’s experimental group. Piglets born to sows in the oxygen-supplementation group (treatment group) were placed in normoxic chambers (≈80 L; FiO_2_ 0.21 ± 0.01), whereas piglets born to control sows were placed in control chambers of the same volume receiving only barn air (FiO_2_ 0.16–0.18). Handling, session duration, and sow–piglet separation were kept consistent among groups to provide similar management conditions. Normoxic chambers were maintained at FiO_2_ at 0.21 ± 0.01 by blending oxygen from an oxygen concentrator (Shenyang Canta Medical Tech. Co., Ltd., Liaoning, China), delivering 98% O_2_, 2 L·min^−1^, with barn air, whereas control chambers received only barn air (FiO_2_ 0.16–0.18). FiO_2_ was continuously monitored, logged every 10 s, and exposures began after steady state (≈3τ; τ = V/Q) was reached. Each session lasted 15 min at 38 ± 1.5 °C, with 4–8 piglets depending on body weight, with handling and sow separation being identical across groups. Humidity was monitored concurrently, and ambient barn FiO_2_ was logged with session means included as covariates in supportive analyses. FiO_2_ analyzers (Maxtec, Salt Lake City, UT, USA) were calibrated daily with room air (20.9%) and checked against a reference gas. Sessions were considered valid if FiO_2_ remained within 0.21 ± 0.01 (normoxia) or 0.16–0.18 (control) for ≥90% of the interval; otherwise, they were flagged for sensitivity analyses.

### 2.4. Data Collection

At parturition, immediately after complete expulsion (within approximately 30–45 s after birth), each piglet was weighed using a digital scale (Super scales and system Co., Ltd., Saraburi, Thailand). For statistical analysis, piglets were classified into three birth-weight categories: Low Birth Weight (LBW; <1.0 kg), Medium Birth Weight (MBW; 1.0–1.3 kg), and High Birth Weight (HBW; >1.3 kg). The status of the umbilical cord (ruptured or intact) was recorded. Blood SpO_2_B was recorded before chamber exposure using a pulse oximeter (VE-H100B, EDAN Instruments Inc., Shenzhen, China), as described previously [27,28]. Subsequently, SpO_2_A was similarly recorded after 15 min of chamber exposure to verify oxygen saturation levels. Blood glucose concentrations were determined using a handheld glucometer (Accu-Chek Instant™, Roche Diagnostics GmbH, Mannheim, Germany). Each piglet was reweighed using the same digital scale 24 h after birth, and their survival status was recorded at 24 h and 3 d, and their weaning weight was recorded at 28 d. At farrowing, data for farrowing duration, average birth interval, total litter size (number born alive, stillbirths, and mummified fetuses) were recorded.

Colostrum intake of piglets was estimated using the following equations:

$$\mathrm{Colostrum}\mathrm{intake}\;(g)=-106+2.26\;\mathrm{WG}+200\;\mathrm{BWB}+0.111\;D-1414\;\frac{WG}{D}+0.0182\;\frac{WG}{BWB}$$

where WG is piglet weight gain over 24 h (g), BWB is birth weight (kg), and D is the duration of colostrum suckling (min) [24].

### 2.5. Statistical Analyses

Descriptive statistics (mean, standard deviation, minimum, and maximum) were generated for all continuous variables, stratified by treatment group, using the PROC MEANS procedure (SAS, version 9.4; SAS Institute Inc., Cary, NC, USA). Correlation analyses (PROC CORR) were performed to evaluate associations among sow reproductive performances and piglets performance variables (Brix, birth interval, Cumulative birth interval, farrowing duration, total born, born alive, mummified, stillbirth, piglet birthweight, SPO_2_B, SPO_2_A, oxygen saturation difference [SPO_2_D], colostrum intake, glucose concentration at 8 h, and weaning weight), both overall and within treatment groups.

Continuous outcomes (SPO_2_B, SPO_2_A, SPO_2_D, colostrum intake, blood glucose at 8 h, and weaning weight) were analyzed using linear mixed-effects models (PROC MIXED) with treatment group as a fixed effect and sow as a random effect. Post-oxygen saturation was analyzed both by repeated-measures modelling (time × group interaction) and by ANCOVA, adjusting post values for baseline (pre-oxygen) values. Covariates included birthweight (or birthweight category), batch and parity; LS-means and adjusted between-group differences with 95% CIs were reported. Assumptions were checked and data transformed if necessary. Categorical variables were analyzed using chi-square tests (PROC FREQ) to examine associations between treatment group, piglet birthweight category, and survival outcomes (24 h and 3 d survival). Piglet birthweight (PBwt) was categorized into three groups for subsequent analyses: ≤1.0 kg (LBW), 1.01–1.30 kg (MBW), and >1.30 kg (HBW). An additional variable (PO_2_D) was computed as the difference between postnatal arterial oxygen saturation at birth and 15 min (PO_2_A − PO_2_B). To account for clustering of piglets within litters, mixed-effects logistic regression models were fitted using the PROC GLIMMIX procedure (SAS software, version 9.4; SAS Institute Inc., Cary, NC, USA). Binary outcomes of survival at 24 h and survival at 3 d were modelled as dependent variables. Fixed effects included treatment group, birthweight category, and their interaction, while sow identity was included as a random effect. Least-squares means with pairwise differences were estimated for each fixed effect. Exploratory dose–response models included session-mean FiO_2_ as a continuous predictor. Effect sizes are presented as adjusted odds ratios or β (95% CIs). Analyses were performed in SAS (V 9.4, Cary, NC, USA).

## 3. Results

**Figure 1:**
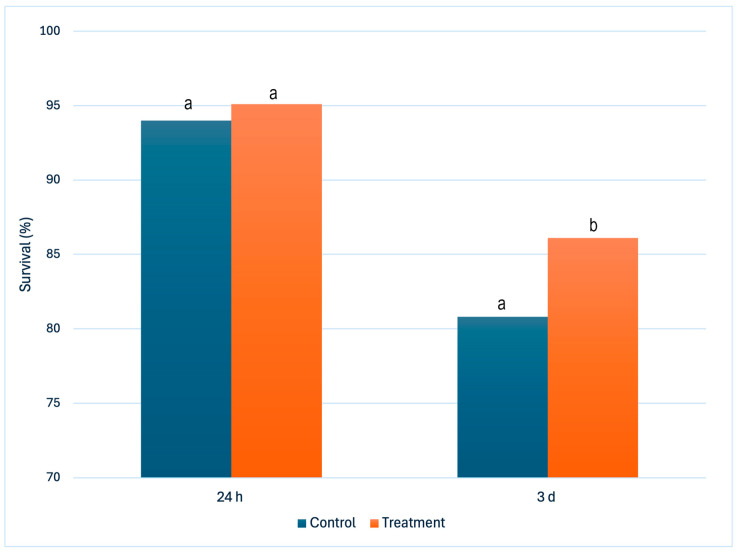
Survival of liveborn piglets at 24 h and 3 d postpartum in control and treatment groups. Bars with different letters differ significantly at *p* < 0.01.

Baseline sow and litter characteristics did not differ between treatment groups, indicating that a balanced allocation was achieved (Table 1). As shown in Table 2, piglet SpO_2_B was higher in the treatment group compared with controls, but the observed difference between groups (<0.3%), while statistically significant, was of negligible biological relevance. At 15 min postpartum (SpO_2_A), oxygen-treated piglets exhibited markedly higher values compared with controls (*p* < 0.0001). Colostrum intake was greater in the treatment group compared with controls (*p* < 0.0001) and, similarly, blood glucose concentration at 8 h postpartum was higher in treated piglets compared with controls (*p* < 0.0001). In addition, weaning weight was greater in the treatment group than in controls (*p* < 0.0001). Overall, at 24 h, survival rates were similar between groups with no statistical difference (Figure 1). However, by 3 d, survival improved in treated piglets (86.1% vs. 80.8%; *p* = 0.005).

**Figure 2:**
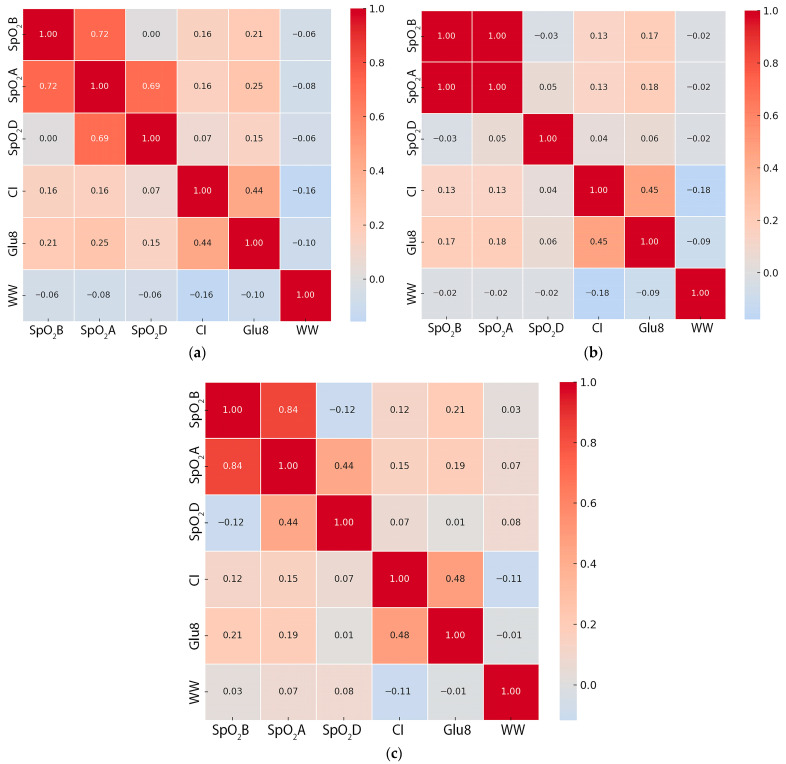
Correlation heatmaps of piglet oxygenation, metabolic, and growth traits. (**a**) Combined dataset (all piglets), (**b**) control group, and (**c**) oxygen-supplemented group. Variables include SpO_2_B (pre-oxygen saturation), SpO_2_A (post-oxygen saturation), PO_2_D (oxygen saturation difference), CI (colostrum intake), Glu8 (blood glucose at 8 h), and WW (weaning weight).

**Figure 3:**
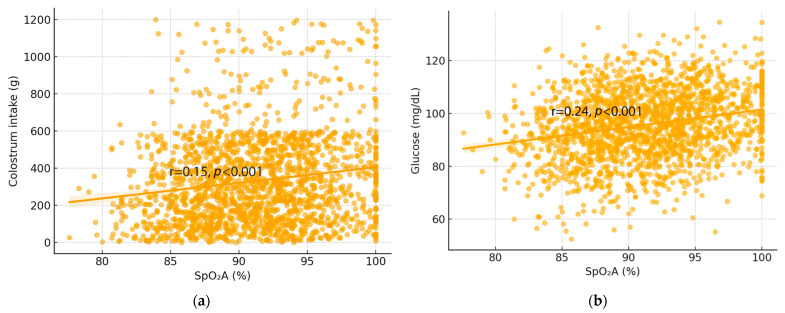
Relationship between SpO_2_A and (**a**) colostrum intake (g) (r = 0.15, R^2^ = 0.02) and (**b**) blood glucose concentration at 8 h postpartum (mg/dL) (r = 0.24, R^2^ = 0.06). The low R^2^ values (0.02–0.06) indicate that oxygen saturation accounts for only a small proportion of variance in colostrum intake and glucose, consistent with weak correlations.

Correlation analysis indicated that SpO_2_A had a correlation with weaning weight and positive relationships with both colostrum intake and 8 h blood glucose concentrations (Figure 2a–c). These associations were observed in both groups but were stronger in oxygen-treated piglets, indicating that improved oxygenation enhanced early energy status. Regression analysis provided additional support for this interpretation, showing that higher SpO_2_A was weakly but positively associated with higher colostrum intake (r = 0.15, *p* < 0.001; Figure 3a) and higher blood glucose concentrations after 8 h (r = 0.24, *p* < 0.001; Figure 3b). Overall, although oxygenation was associated with improvements in early physiological variables, the magnitude of these correlations likely suggests only limited biological influence.

**Figure 4:**
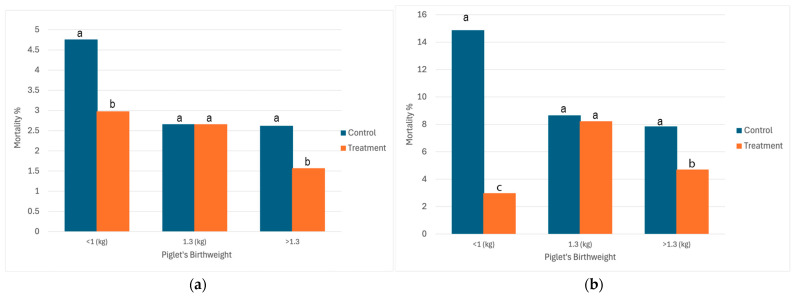
Mortality rate by piglet birthweight category (Control and Treatment) at (**a**) 24 h and (**b**) 3 d. Bars with different letters differ at *p* < 0.05.

Oxygen-treated piglets showed a significant reduction in mortality compared with controls across specific birth-weight categories (Figure 4). The most pronounced effect was observed in the LBW piglets (<1.0 kg), where 24 h mortality declined from 4.8% in the control group to 3.0% in the treatment group (*p* < 0.05). A similar reduction was found in HBW piglets (>1.3 kg), with mortality decreasing from 2.9% to 1.6% (*p* < 0.05). In contrast, MBW piglets (1.0–1.3 kg) showed no difference between groups (2.7% in both) (Figure 4a). A similar pattern was observed at 3 d (Figure 4b). The LBW piglets benefited the most from oxygen supplementation, showing a reduction in mortality from 14.9% in controls to 2.98% in the treated group (*p* < 0.0001). Among MBW piglets, mortality was not affected by treatment. In HBW piglets (>1.3 kg), oxygen supplementation resulted in a decline in mortality (7.85% vs. 4.71%; *p* < 0.05).

## 4. Discussion

Effects of oxygen treatment were observed in this study, although the magnitude and level of significance were dependent on piglet birth weights. Our analysis indicated that this variation was not attributable to unequal sample sizes but rather to inherent physiological differences among weight groups. MBW piglets generally displayed stable postnatal adaptation, limiting the detectable treatment response, while heavier piglets were more likely to experience transient hypoxia during farrowing, resulting in a modest improvement following oxygen supplementation. The beneficial response to normoxic oxygen supplementation was most pronounced among the LBW piglets, the improvement likely reflecting enhanced oxygen delivery and aerobic metabolism during a critical transition period when metabolic reserves are limited [12,13,29]. Conversely, larger piglets, often born later in the farrowing sequence, are more susceptible to transient hypoxia caused by umbilical cord compression or delayed breathing [20,27] and, although speculative in the absence of specific data, it is possible that larger piglets would suffer a more prolonged delivery. Oxygen supplementation facilitated faster reoxygenation and metabolic recovery in these individuals. MBW piglets will likely have greater physiological reserves at birth than do LBW littermates, such as improved thermoregulatory capacity, increased energy availability (glycogen and muscle mass), improved competitiveness for teats, and more effective colostrum intake and so passive immunity transfer [4,14]. These characteristics, together with a lower surface area to volume ratio, promote a more sustained postnatal adaptation, reducing the observable treatment response in this weight range. As such, mortality among MBW piglets may be more related to postnatal challenges such as intra-litter competition, starvation, or being overlaid, which are less influenced by immediate oxygenation status at birth [30,31,32]. This may explain why normoxic oxygen supplementation did not improve survival in MBW piglets despite clear benefits among both LBW and HBW piglets, where the risk of hypoxia during farrowing and immediately postpartum is more pronounced and thus more susceptible to a hypoxia-targeted intervention.

The present study revealed that oxygen supplementation in the early postnatal period improved piglet oxygen saturation, colostrum intake, blood glucose concentration, survival at 3 d, and weaning weight. The treated piglets’ higher average weaning weight probably resulted from initial improved teat access and suckling activity at birth, which encourages higher milk and colostrum consumption and supports higher circulating early glucose concentrations. While there was no meaningful difference in piglet survival at 24 h, by 3 d and continuing until day 21, there was a notable benefit to oxygen therapy, highlighting the significance of early intervention in determining longer-term results. The 15 min exposure period was sufficient for neonatal piglets to achieve stable oxygen saturation, consistent with previous physiological data in piglets and human neonates [20,21,23,33]. Mechanistically, adequate oxygen availability supports ATP generation, sustaining vital functions such as cardiac activity, muscle contraction, and organ development. Consistent with previous studies, oxygen-supplemented piglets also displayed greater livability than controls, reflecting enhanced oxidative metabolism and improved energy availability at birth [33]. Together, these findings highlight the potential of oxygen therapy to deliver measurable benefits in commercial pig production, even when the immediate effects appear small.

During the transition from the fetal to the neonatal stage, piglets are highly vulnerable to oxygen instability due to prolonged farrowing, immature cardiopulmonary systems, and limited oxygen reserves at birth, which predispose them to hypoxic stress and force a metabolic shift from aerobic to anaerobic pathways. This shift is characterized by lactate accumulation, reduced ATP production, and an energy deficit that impairs thermoregulation, neuromuscular reflexes, and metabolic vitality [33]. Oxygen therapy enhances neonatal survival by promoting ATP synthesis and reducing lactate accumulation, which in turn supports reflex activity, effective suckling, and colostrum intake, thereby contributing to improved glucose regulation and passive immunity [20]. However, excess oxygen supplements can lead to oxidative stress [18], which emphasizes how crucial it is to keep oxygen levels within a physiologically appropriate range. These adaptive processes are especially important for compromised low-birth-weight piglets and intra-uterine growth restriction piglets, which show high rates (70–90%) of inadequate colostrum intake even under optimal maternal conditions [34]. The observation that oxygen-treated piglets consume more colostrum suggests that this intervention could be particularly beneficial for LBW neonates, assisting them in overcoming early competitive disadvantages. Therefore, oxygen therapy should be viewed as a targeted approach for at-risk piglets, where minor physiological advantages may result in significant gains in development and survival, rather than as a general intervention.

In human neonatology, oxygen therapy is a cornerstone of resuscitation for preterm and hypoxic infants. While its life-saving role is certain, decades of research have emphasized the importance of careful titration to minimize oxidative stress [35,36]. Early randomized trials demonstrated that resuscitation with room air achieved outcomes comparable to 100% oxygen while reducing oxidative injury, establishing the principle that more oxygen is not necessarily better and forming the basis for targeted oxygen strategies [23]. Applied to swine systems, this principle highlights that oxygen should be administered at physiological levels rather than through indiscriminate high-dose supplementation. Most swine research has focused on other strategies to improve neonatal survival, including colostrum supplementation and glucose administration [37], while oxygen therapy has received comparatively little attention. Existing evidence shows that oxygen inhalation at birth can reduce early mortality [33], with more recent field studies reporting improved glucose concentrations, colostrum intake, and survival [20], whereas prolonged farrowing has been associated with placental complications, stillbirths, and fetal hypoxia [38]. Although previous swine research was restricted to small experimental cohorts under carefully controlled conditions, it provided valuable physiological information [20,33]. In contrast, the current study assessed almost 1800 piglets under commercial farm management, making it one of the largest datasets to date. It also provides strong evidence that oxygen given within physiological restrictions can enhance colostrum intake, metabolic stability, and survival rate. These enhancements have a biological basis in increased ATP availability, which promotes organ function, reflex activity, and thermoregulation. Increased vitality promotes the consumption of colostrum, and stable glucose balance lowers hypoglycemia, both of which support early growth. The cumulative benefits of oxygen supplementation, which support metabolic stability, food acquisition, result in increased weaning weights.

The practical implications of oxygen supplementation are noteworthy for commercial swine production. Previous studies have demonstrated that improvements in early survival and growth can influence overall herd performance under commercial conditions [30,39,40]. In this context, the present findings highlight the biological relevance of improving early piglet viability and survival, rather than emphasizing fixed numerical or economic gains. Even relatively small physiological improvements at birth may accumulate across cohorts and contribute to improved animal welfare and biological efficiency at the herd level [14,41]. Although a formal economic or cost–benefit analysis was not conducted, the operational cost of oxygen supplementation in the present study was approximately 0.6 THB per piglet, based on consumable and operational expenses under the specific farm conditions evaluated. This estimate is context-specific and may vary depending on farm scale, equipment, and management practices; therefore, broader economic conclusions cannot be drawn from the present data.

Though this study provides valuable information about the beneficial effects of oxygen supplementation in commercial swine production, its potential limitations should be acknowledged. These include the absence of mechanistic biomarkers such as lactate, cortisol, or body temperature, which would have provided further insights into the physiological pathways of improved survival. Nevertheless, the consistent increases in SpO_2_, glucose, and colostrum intake strongly indicate enhanced aerobic metabolism. Although there were evident increases in survival and weaning weight, the lack of mechanistic biomarkers, such as blood lactate, limits the capacity to completely explain the physiological pathways underlying the observed positive outcomes. Despite being statistically significant, the effect size was small and can differ from farm to farm according to environmental factors, sow genetics, and management techniques. Lastly, the follow-up duration was set at 21 d, making it impossible to assess longer-term results, including growth to market weight, feed conversion efficiency, or reproductive performance.

This study reveals the biological relevance of oxygen supplementation in neonatal piglets. However, several crucial areas still require further research before they can be widely implemented in commercial swine production. Establishing accurate dose–response protocols, including the optimal timing, concentration, and duration of oxygen administration, should be a focus for further development in livestock production. To identify the best integrated approaches for enhancing neonatal outcomes, future research should compare the effects of oxygen treatment with other supportive interventions, including colostrum enhancers, oral glucose supplements, and thermal support. Additionally, economic modeling, specifically cost-effectiveness analyses, will assist in clarifying the balance between the cost of oxygen delivery systems and the financial benefits that accrue from increased growth and survival. It will also be necessary to validate across several farms and management systems to ensure practical relevance, expansion, and consistency in commercial swine production.

## 5. Conclusions

Piglet survival and early growth under commercial conditions are improved by the application of oxygen supplementation at birth. With data from over 1800 piglets, this study provides solid evidence that even small effects can have significant physiological and financial advantages. Integration of cost–benefit analyses and refined protocols would enhance the effectiveness of oxygen supplementation as a strategy to improve welfare and productivity by enhancing colostrum intake, growth and survival in commercial swine production. Oxygen supplementation reduced early mortality in LBW and HBW piglets, but no clear benefit was observed in MBW piglets, which are generally more stable at birth.”

## Figures and Tables

**Table 1:** Summary of sow reproductive performances (Control vs. Treatment).

Variables	Control (*n* = 48)		Treatment (*n* = 47)		*p*-Value
	Means ± SD	Range	Means ± SD	Range	
Parity (*n*)	3.8 ± 1.9	1–7	3.6 ± 1.8	1–7	0.59
Brix (%)	24.6 ± 2.1	20.4–29.3	24.8 ± 1.8	21.1–30.1	0.88
Birth interval	4.9 ± 2.6	3.24–5.73	4.8 ± 0.5	3.62–6.09	0.41
Cumulative birth interval	46.5 ± 29.3	29.04–60.9	46.9 ± 6.8	36.35–65.78	0.56
Farrowing Time (min)	199.49 ± 44.7	125–314	195.6 ± 44.1	125–286	0.68
Total born (*n*)	19.2 ± 1.6	15–23	19.7 ± 1.4	16–23	0.06
Born Alive (*n*)	17.1 ± 1.3	14–20	17.9 ± 1.4	15–20	0.01 *
Mummified (*n*)	0.8 ± 0.9	0–3	0.6 ± 0.8	0–3	0.25
Stillbirth (*n*)	1.2 ± 1.3	0–5	1.3 ± 1	0–4	0.77
Birth weight (kg)	1.2 ± 0.1	0.7–1.6	1.1 ± 0.1	0.7–1.6	0.49

* Statistically significant difference (*p* < 0.05), *n* indicates the number of sows (unit of randomization).

**Table 2:** Oxygen supplementation and its effects on piglet physiology and performance.

Variable	Control (914)	Treatment (923)	*p*-Value
SpO_2_B (%)	87.6 ± 3.5	87.9 ± 3.3	<0.0001
SpO_2_A (%)	88.1 ± 3.5	94.2 ± 3.6	<0.0001
Colostrum intake (g)	319.5 ± 233.4	348.4 ± 262.5	<0.0001
Glucose (mg/dL)	93.8 ± 13.1	97.7 ± 13.0	<0.0001
Weaning weight (kg)	6.6 ± 0.7	6.9 ± 0.8	<0.0001

## Data Availability

The data that support the findings of this study are available from the corresponding author upon reasonable request.
